# Treatment outcomes of T and natural‐killer/T‐cell lymphoma with ifosfamide, carboplatin and etoposide chemotherapy

**DOI:** 10.1002/cnr2.1552

**Published:** 2022-04-28

**Authors:** Tricia Tay, Nagavalli Somasundaram, Cindy Lim, Lay Poh Khoo, Allan Zhi Kai Goh, Yuh Shan Lee, Xin Liu, Miriam Tao, Richard Quek, Mohamad Farid, Eileen Poon, Jason Y. S. Chan, Esther W. Y. Chang, Valerie S. W. Yang, Yeow Tee Goh, Daryl Tan, Colin Diong, Nicholas F. Grigoropoulos, Chandramouli Nagarajan, Michelle Poon, Sanjay de Mel, Anand Jeyasekharan, Esther H. L. Chan, Joanne Lee, Yen Lin Chee, Soon Thye Lim, Tiffany Tang

**Affiliations:** ^1^ Division of Medical Oncology National Cancer Centre Singapore Singapore Singapore; ^2^ Division of Clinical Trials & Epidemiological Sciences National Cancer Centre Singapore Singapore Singapore; ^3^ Department of Haematology Singapore General Hospital Singapore Singapore; ^4^ Department of Haematology‐Oncology National University Hospital Singapore Singapore Singapore

**Keywords:** Asia, chemotherapy, haematology–oncology, natural‐killer/T‐cell lymphoma, T‐cell lymphoma

## Abstract

**Background:**

Contemporary data of peripheral T‐cell lymphoma (PTCL) and natural‐killer/T‐cell lymphoma (NKTL) patients treated with ifosfamide, carboplatin and etoposide (ICE) are limited.

**Aims:**

We performed a retrospective analysis to estimate outcomes of ICE‐treated PTCL and NKTL patients at three tertiary cancer centres in Singapore.

**Methods and Results:**

Patients were identified through lymphoma databases from National Cancer Centre Singapore (NCCS), National University Hospital, Singapore (NUHS), and Singapore General Hospital (SGH). Responses and survival outcomes were determined from electronic medical records. A total of 75 patients with a median age of 50 were included. ICE was used as first‐line treatment in 14 patients (19%) and as subsequent lines of treatment in 61 patients (81%). The overall response rates (ORR) for all patients was 63% (40% complete response [CR]). The ORR and CR in the first line were 86% and 64% respectively. At a median follow‐up duration of 71.0 months, the median progression‐free (PFS) and overall survival (OS) for all patients were 4.4 months (95%CI, 2.7–6.0) and 16 months (95%CI, 8.3–45.4) respectively.

**Conclusion:**

In summary, ICE showed high ORR but poor PFS in relapsed/refractory PTCL and NKTL. ORR of ICE in the first line setting appears better than real‐world CHOP data and warrants further study.

## 1 INTRODUCTION

Peripheral T‐cell lymphoma (PTCL) and natural‐killer/T‐cell lymphoma (NKTL) represent 10–15% of non‐Hodgkin lymphomas(NHL), with treatment outcomes that generally lag behind their B‐cell counterparts.^1^, ^2^, ^3^ CHOP‐based regimen (cyclophosphamide, doxorubicin, vincristine and prednisolone) is most commonly used in the first line setting in the treatment of PTCL^3^ and L‐asparaginase‐based chemotherapy regimens are generally used in the treatment of NKTL to bypass the multidrug resistant P‐glycoproteins found on NK cells.^4^, ^5^ Ifosfamide, carboplatin and etoposide (ICE) is a commonly used salvage chemotherapy regimen for PTCL and NKTL.^6^ Historically, this regimen produces response rates of about 54%.^6^ In Singapore, ICE has been used both to treat PTCL and NKTL in the first line and relapsed or refractory setting. The purpose of this study is thus to explore the treatment outcomes of PTCL and NKTL patients treated with this regimen from three tertiary academic centres in Singapore.

## 2 METHODS

This is a retrospective observational study using prospectively maintained lymphoma databases from the National Cancer Centre Singapore (NCCS), National University Hospital, Singapore (NUHS), and Singapore General Hospital (SGH). The study was approved by the individual institutional review boards. Patients with histologically confirmed PTCL or NKTL, treated with at least one cycle of ICE in the first line, relapsed or refractory setting between 2001 and 2017 were included in the analysis. Patients with refractory disease had disease that progressed during or within 6 months of completion of their prior treatment regimen. Data on patients' clinical characteristics, treatment and survival outcomes were collected from databases and confirmed using electronic medical records. Patients with composite lymphomas, those who received ICE chemotherapy as part of mobilisation for high‐dose chemotherapy/autologous stem cell transplantation (HDC/ASCT) and those who received concurrent ICE and radiotherapy were excluded from the analysis.

The overall response rates (ORR) (complete response [CR] and partial response [PR]) were determined from physician's assessments in their electronic medical records and confirmed by assessing imaging, bone marrow or biopsy results. Progression‐free survival (PFS) was defined as the interval from first dose of ICE to disease progression or death from any cause. Overall survival (OS) was defined as the interval from first dose of ICE to death from any cause or last follow‐up. Survival probabilities and the median survival times were estimated using the Kaplan–Meier method. Differences between groups were tested using the log‐rank test. Univariable and multivariable analyses were performed using the Cox proportional hazards model. Variable selection was performed using the best subsets method by optimising the Akaike Information Criterion (AIC). The following variables were used in the selection procedure: age, gender, race, histology, stage, B symptoms, extranodal involvement and achievement of CR. LDH and ECOG performance status were not included due to a high percentage of missing data and IPI was not included as its components were already included. Treatment setting was forced in the model and not subjected to variable selection.

A two‐sided *p*‐value of less than .05 was considered statistically significant. All analyses were performed in Stata/SE 15.0 (StataCorp, College Station, Texas).

## 3 RESULTS

A total of 75 patients were included in our study. The median age of patients was 50 (range 21–71), and 58 (77%) were male. Most patients were of Chinese ethnicity (77%), reflecting the general ethnic distribution in Singapore. The histological subsets included PTCL‐not otherwise specified (NOS) (35%), angioimmunoblastic T‐cell lymphoma (AITL) (23%), NKTL (20%), anaplastic large cell lymphoma (17%), and others including monomorphic enteropathic intestinal T‐cell lymphoma (MEITL),^7^, ^8^ previously known as type II enteropathic T‐cell lymphoma, and hepatosplenic T‐cell lymphoma, which made up 5% of the tumour subsets. At diagnosis, 80% of patients had advanced‐stage disease, 21% had B symptoms and 77% had disease involvement of extranodal sites. Eastern Cooperative Oncology Group (ECOG) performance status was 0 or 1 in 75% of patients, and unknown in about 23%.(Table 1). Using the reverse Kaplan–Meier method, the median follow‐up time was 71.0 months (95% CI, 50.0–103.4 months) or 5.9 years (95% CI, 4.2–8.6 years).

**TABLE 1:** Demographics and clinical characteristics

Non‐NKTCL						NKTCL				
Characteristic	Total (n = 60)	First line (n = 7)	Refractory (n = 33)	Relapsed (n = 20)	*p*‐value	Total (n = 15)	First line (n = 7)	Refractory (n = 6)	Relapsed (n = 2)	*p*‐value
Age at ICE treatment, years										
Median (range)	51.5 (21–71)	55 (26–62)	52 (21–71)	48 (21–70)	.826	49 (25–68)	51 (36–68)	45 (25–53)	43 (37–49)	.367
≤ 60	47 (78.3)	5 (71.4)	26 (78.8)	16 (80.0)	.819	13 (86.7)	5 (71.4)	6 (100)	2 (100)	.600
> 60	13 (21.7)	2 (28.6)	7 (21.2)	4 (20.0)		2 (13.3)	2 (28.6)	0 (0)	0 (0)	
Gender					**.043**					.723
Male	46 (76.7)	3 (42.9)	25 (75.8)	18 (90.0)		12 (80.0)	6 (85.7)	4 (66.7)	2 (100)	
Female	14 (23.3)	4 (57.1)	8 (24.2)	2 (10.0)		3 (20.0)	1 (14.3)	2 (33.3)	0 (0)	
Race					.994					1.000
Chinese	45 (75.0)	6 (85.7)	25 (75.8)	14 (70.0)		13 (86.7)	5 (71.4)	6 (100)	2 (100)	
Indian	5 (8.3)	0 (0)	3 (9.1)	2 (10.0)		0 (0)	0 (0)	0 (0)	0 (0)	
Malay	4 (6.7)	0 (0)	2 (6.1)	2 (10.0)		1 (6.7)	1 (14.3)	0 (0)	0 (0)	
Others	6 (10.0)	1 (14.3)	3 (9.1)	2 (10.0)		1 (6.7)	1 (14.3)	0 (0)	0 (0)	
Histology					.155					
PTCL‐NOS	26 (43.3)	4 (57.1)	17 (51.5)	5 (25.0)						
ALCL	13 (21.7)	1 (14.3)	6 (18.2)	6 (30.0)				NA		
AITL	17 (28.3)	1 (14.3)	7 (21.2)	9 (45.0)						
EATL, HSTL	4 (6.7)	1 (14.3)	3 (9.1)	0 (0)						
Stage at diagnosis					**<.001**					.089
1	2 (3.3)	1 (14.3)	0 (0)	1 (5.0)		1 (6.7)	0 (0)	0 (0)	1 (50.0)	
2	8 (13.3)	4 (57.1)	0 (0)	4 (20.0)		3 (20.0)	0 (0)	3 (50.0)	0 (0)	
3	15 (25.0)	0 (0)	8 (24.2)	7 (35.0)		1 (6.7)	1 (14.3)	0 (0)	0 (0)	
4	35 (58.3)	2 (28.6)	25 (75.8)	8 (40.0)		10 (66.7)	6 (85.7)	3 (50.0)	1 (50.0)	
B symptoms					**.022**					.790
Yes	10 (16.7)	3 (42.9)	2 (6.1)	5 (25.0)		6 (40.0)	2 (28.6)	3 (50.0)	1 (50.0)	
No	47 (78.3)	4 (57.1)	30 (90.9)	13 (65.0)		9 (60.0)	5 (71.4)	3 (50.0)	1 (50.0)	
Unknown	3 (5.0)	0 (0)	1 (3.0)	2 (10.0)		0 (0)	0 (0)	0 (0)	0 (0)	
LDH					.214					.400
Elevated	25 (41.7)	2 (28.6)	15 (45.5)	8 (40.0)		2 (13.3)	1 (14.3)	0 (0)	1 (50.0)	
Normal	12 (20.0)	3 (42.9)	4 (12.1)	5 (25.0)		3 (20.0)	3 (42.9)	0 (0)	0 (0)	
Unknown	23 (38.3)	2 (28.6)	14 (42.4)	7 (35.0)		10 (66.7)	3 (42.9)	6 (100)	1 (50.0)	
Extranodal involvement					.820					.133
Yes	44 (73.3)	6 (85.7)	25 (75.8)	13 (65.0)		14 (93.3)	7 (100)	6 (100)	1 (50.0)	
No	13 (21.7)	1 (14.3)	7 (21.2)	5 (25.0)		1 (6.7)	0 (0)	0 (0)	1 (50.0)	
Unknown	3 (5.0)	0 (0)	1 (3.0)	2 (10.0)		0 (0)	0 (0)	0 (0)	0 (0)	
ECOG performance status					.583					1.000
0	38 (63.3)	6 (85.7)	18 (54.5)	14 (70.0)		6 (40.0)	3 (42.9)	2 (33.3)	1 (50.0)	
1	11 (18.3)	1 (14.3)	8 (24.2)	2 (10.0)		1 (6.7)	1 (14.3)	0 (0)	0 (0)	
2	2 (3.3)	0 (0)	2 (6.1)	0 (0)		0 (0)	0 (0)	0 (0)	0 (0)	
Unknown	9 (15.0)	0 (0)	5 (15.2)	4 (20.0)		8 (53.3)	3 (42.9)	4 (66.7)	1 (50.0)	
IPI					.553					NA
Low	7 (11.7)	2 (28.6)	2 (6.1)	3 (15.0)		1 (6.7)	1 (14.3)	0 (0)	0 (0)	
Low‐intermediate	3 (5.0)	1 (14.3)	1 (3.0)	1 (5.0)		0 (0)	0 (0)	0 (0)	0 (0)	
High‐intermediate	10 (16.7)	1 (14.3)	7 (21.2)	2 (10.0)		1 (6.7)	1 (14.3)	0 (0)	0 (0)	
High	3 (5.0)	1 (14.3)	2 (6.1)	0 (0)		0 (0)	0 (0)	0 (0)	0 (0)	
Unknown	37 (61.7)	2 (28.6)	21 (63.6)	14 (70.0)		13 (86.7)	5 (71.4)	6 (100)	2 (100)	

*Note*: Data presented as No. (%) unless otherwise indicated.

Abbreviation: IPI, International Prognostic Index.

In this study, ICE was used as first‐line treatment in 14 patients (19%) and as subsequent lines of treatment in 61 patients (81%). Among those who received ICE as first line treatment, 50% had NKTL and 29% had PTCL‐NOS. Among the 61 patients who received ICE as subsequent treatment, 64% had disease refractory to their prior treatment. The patients received a median of 3 cycles of ICE. Patients treated in first line received more cycles of ICE than those with relapsed/refractory disease (median of 6 versus 3).

The ORR for all patients 62.7% (40% CR and 22.7% PR). Among patients who were treated with ICE in first line, relapsed, and refractory setting, the ORR were 85.7%, 68.2% and 51.3% and the CR rates were 64.3%, 45.5% and 28.2% respectively. The median PFS and OS for all patients was 4.4 months (95% confidence interval [CI], 2.7–6.0) and 16 months (95% CI, 8.3–45.4) respectively. Among patients who were treated with ICE in first line, relapsed, and refractory setting, the median PFS were not reached, 4.4 and 3.4 months respectively. The median OS were 51.9, 33.9 and 10.9 months for those treated in the first line, relapsed, and in the refractory setting.(Table 2) For those who achieved a CR (N = 30) and PR (N = 17) on ICE, the median PFS was 17.9 months (95% CI, 6.3 to 50.3 months) and 4.6 months (95% CI, 1.7 to 18.8 months) respectively.

**TABLE 2:** Treatment outcomes

Non‐NKTCL						NKTCL				
	Total (n = 60)	First line (n = 7)	Refractory (n = 33)	Relapsed (n = 20)	*p*‐value	Total (n = 15)	First line (n = 7)	Refractory (n = 6)	Relapsed (n = 2)	*p*‐value
Median no. of cycles (range)	3 (1–6)	6 (4–6)	3 (1–4)	3 (1–5)	**<.001**	3 (1–7)	4 (1–7)	2 (1–3)	4.5 (3–6)	.168
Best response (%)					.149					.640
CR	23 (38.3)	6 (85.7)	9 (27.3)	8 (40.0)		7 (46.7)	3 (42.9)	2 (33.3)	2 (100)	
PR	14 (23.3)	1 (14.3)	8 (24.2)	5 (25.0)		3 (20.0)	2 (28.6)	1 (16.7)	0 (0)	
SD	4 (6.7)	0 (0)	4 (12.1)	0 (0)		0 (0)	0 (0)	0 (0)	0 (0)	
PD	16 (26.7)	0 (0)	10 (30.3)	6 (30.0)		4 (26.7)	1 (14.3)	3 (50.0)	0 (0)	
Not done/unknown	3 (5.0)	0 (0)	2 (6.1)	1 (5.0)		1 (6.7)	1 (14.3)	0 (0)	0 (0)	
Progression‐free survival										
No. of events/No. of patients	49/60	1/7	30/33	18/20		12/15	5/7	6/6	1/2	
Median PFS, months (95% CI)	4.6 (2.3, 7.4)	Not reached	3.4 (1.5, 5.3)	4.4 (1.6, 23.9)	**.004**	4.2 (1.0, 5.8)	5.1 (1.0, UD)	1.4 (0.6, UD)	0.4 (0.4, UD)	.393
6‐month PFS rate, % (95% CI)	42.6 (29.9, 54.7)	100	30.3 (15.9, 46.1)	45.0 (23.1, 64.7)		22.2 (5.5, 45.9)	34.3 (4.8, 68.6)	0	50.0 (0.6, 91.0)	
Overall survival										
No. of events/No. of patients	38/60	0/7	26/33	12/20		11/15	5/7	5/6	1/2	
Median OS, months (95% CI)	20.3 (10.8, 79.3)	Not reached	13.9 (6.9, 24.6)	33.9 (3.9, UD)	**.024**	6.7 (1.4, 33.1)	8.2 (1.0, UD)	5.2 (0.6, UD)	33.1 (33.1, UD)	.077
12‐month OS rate, % (95% CI)	59.0 (45.3, 70.4)	100	51.0 (33.0, 66.5)	59.6 (35.1, 77.4)		40.0 (14.9, 64.3)	34.3 (4.8, 68.6)	22.2 (1.0, 61.5)	100	

Abbreviations: CR, complete response; OS, overall survival; PD, progression of disease; PFS, progression free survival; PR, partial response; SD, stable disease; UD, undefined.

Univariable analyses revealed that patients treated in the relapsed or refractory setting, high‐intermediate (HI) or high (H) IPI scores, advanced‐stage disease, and not achieving a CR with treatment, were associated with poorer PFS. Histological subtypes of NKTL, EATL, and hepatosplenic T‐cell lymphoma (in contrast to PTCL‐NOS, AITL and ALCL), advanced stage, HI or H IPI scores, and not achieving CR were associated with a poorer OS. On multivariable analyses, after adjusting for treatment setting, histological subtypes of NKTL, EATL and hepatosplenic T‐cell lymphoma, advanced‐stage disease and not achieving CR to ICE were associated with a poorer PFS and OS. Additionally, receiving treatment after age of 60, presence of B symptoms and extranodal involvement were other factors that were associated with a poorer OS on multivariable analyses (Tables 3 and 4).

**TABLE 3:** Univariable and multivariable analysis of progression‐free survival

Variable	No. of events/patients	Univariable analysis		Multivariable analysis^a^	
		Hazard ratio (95% CI)	*p‐*value	Hazard ratio (95% CI)	*p‐*value
Treatment setting			.004		.031
First line	6/14	1		1	
Refractory	36/39	3.68 (1.54, 8.81)		3.38 (1.20, 9.48)	
Relapsed	19/22	2.56 (1.02, 6.42)		3.58 (1.17, 11.01)	
Age at ICE treatment, years			.459		
≤60	49/60	1			
>60	12/15	1.28 (0.68, 2.42)			
Gender			.160		
Male	50/58	1			
Female	11/17	0.64 (0.33, 1.23)			
Race			.502		
Chinese	50/58	1			
Non‐Chinese	11/17	0.80 (0.42, 1.55)			
Histology			.527		.031
Non‐NKTCL	49/60	1		1	
NKTCL	12/15	1.23 (0.65, 2.33)		2.61 (1.09, 6.21)	
Stage at diagnosis			.004		.057
1–2	8/14	1		1	
3–4	53/61	2.72 (1.28, 5.79)		2.29 (0.92, 5.70)	
B symptoms			.183		
Yes	10/16	0.64 (0.32, 1.28)			
No	48/56	1			
LDH			.068		
Elevated	24/27	2.04 (0.91, 4.55)			
Normal	8/15	1			
Extranodal involvement			.275		
Yes	47/58	1.43 (0.74, 2.76)			
No	11/14	1			
ECOG performance status			.118		
0–1	43/56	1			
2	2/2	4.09 (0.92, 18.15)			
IPI			.019		
Low, low‐intermediate	5/11	1			
High‐intermediate, high	12/14	3.34 (1.15, 9.71)			
Complete response (CR)			<.001		.001
No	41/45	3.76 (2.13, 6.63)		2.76 (1.52, 5.00)	
Yes	20/30	1		1	

*Note*: For multivariable analysis, all variables were included in the best subsets selection procedure except for LDH, ECOG performance status and IPI. Treatment setting was forced in the model and not subjected to variable selection.

^a^ No. of events/patients for multivariable analysis = 57/71.

**TABLE 4:** Univariable and multivariable analysis of overall survival

Variable	No. of events/patients	Univariable analysis		Multivariable analysis^a^	
		Hazard ratio (95% CI)	*p*‐value	Hazard ratio (95% CI)	*p‐*value
Treatment setting			.068		.016
First line	5/14	1		1	
Refractory	31/39	2.35 (0.91, 6.08)		5.78 (1.60, 20.83)	
Relapsed	13/22	1.33 (0.47, 3.76)		5.22 (1.31, 20.75)	
Age at ICE treatment, years			.192		.040
≤60	39/60	1		1	
>60	10/15	1.64 (0.81, 3.32)		2.65 (1.10, 6.38)	
Gender			.393		
Male	40/58	1			
Female	9/17	0.74 (0.35, 1.52)			
Race			.525		
Chinese	41/58	1			
Non‐Chinese	8/17	0.79 (0.37, 1.68)			
Histology			.101		.001
Non‐NKTCL	38/60	1		1	
NKTCL	11/15	1.82 (0.92, 3.60)		6.29 (2.26, 17.48)	
Stage at diagnosis			.002		.001
1–2	4/14	1		1	
3–4	45/61	3.92 (1.40, 10.96)		5.71 (1.73, 18.89)	
B symptoms			.522		.013
Yes	10/16	1.27 (0.63, 2.56)		3.04 (1.31, 7.06)	
No	37/56	1		1	
LDH			.229		
Elevated	19/27	1.72 (0.69, 4.32)			
Normal	7/15	1			
Extranodal involvement			.055		.014
Yes	41/58	2.16 (0.91, 5.12)		2.90 (1.14, 7.35)	
No	6/14	1		1	
ECOG performance status			.981		
0–1	34/56	1			
2	1/2	1.02 (0.14, 7.54)			
IPI			.015		
Low, low‐intermediate	4/11	1			
High‐intermediate, high	11/14	3.91 (1.20, 12.71)			
Complete response (CR)			<.001		<.001
No	35/45	3.82 (1.96, 7.46)		5.80 (2.60, 12.92)	
Yes	14/30	1		1	

*Note*: For multivariable analysis, all variables were included in the best subsets selection procedure except for LDH, ECOG performance status and IPI. Treatment setting was forced in the model and not subjected to variable selection.

^a^ No. of events/patients for multivariable analysis = 46/71.

## 4 DISCUSSION

The ICE regimen was developed as a salvage regimen for relapsed and refractory lymphomas. In this study, we show that patients with relapsed and refractory PTCL and NKTL treated with ICE have relatively high ORR (68% and 51% respectively), but the CR rates were poor (45% and 28%) and the PFS were short (4.4 and 3.4 months) respectively. These results are comparable to previously reported results of ICE in PTCL where ORR were 54% (PR 23%, CR 31%). Novel combinations with ICE should be explored to improve the CR rates and durability of response in R/R PTCL, especially since such patients require an effective bridging regiment to HDC/ASCT. Some novel agents that have been combined with ICE include romidepsin^9^ and selinexor (NCT03212937), and further studies will reveal if these novel combinations are superior to ICE in R/R PTCL.

CHOP is the most commonly used chemotherapy regimen in the first line treatment of PTCL and brentuximab vedotin (BV)‐CHP is now used to treat CD30‐positive PTCL.^10^There is no standard of care for NKTL. In the localised setting, regimens such as DeVIC, ICE or L asparaginase based regimens such as SMILE, GELOX are considered to be effective and acceptable treatment options. In the advanced setting, L asaparaginase based regimens are considered in the first line setting.^11^ In our study, ICE was used in the first line setting for 14 PTCL and NKTL patients and the ORR was 85.7% (CR 64.3%). These results appear better than real‐world data for CHOP, where expected ORR are about 65% and 25% of patients have refractory disease.^12^

While the study is limited by the retrospective nature of the analyses and the small numbers of the individual subtypes of PTCL, to our knowledge, this study the provides the largest data on the outcomes of PTCL and NKTL patients treated with ICE in the first line and relapsed/refractory setting. Results from this study will help to evaluate our current treatment strategies and plan future clinical trials for this group of diseases.

In conclusion, when ICE is used to treat relapsed/refractory PTCL and NKTL, response rates are relatively high but duration of response is poor. Future investigation should evaluate the role of novel agents in combination with ICE to improve current treatment outcomes. ICE appears to have better response rates compared with CHOP in the first line treatment of PTCL and should be evaluated further as a potential first line treatment for this group of diseases.

## CONFLICT OF INTEREST

The authors have stated explicitly that there are no conflicts of interest in connection with this article.

## AUTHOR CONTRIBUTIONS

T. T., N. S., and T. T. contributed equally to the study design, acquisition and interpretation of the data, and drafting the manuscript. C. L. performed statistical analysis of the data. L. P. K., A. Z. K. G., and X. L. helped to collect data. Y. S. L., M. T., R. Q., M. F., E. P., J. Y. S. C., E. W. Y. C., V. S. W. Y., Y. T. G., D. T., C. D., N. F. G., C. N., M. P., S. d. M., A. J., E. H. L. C., J. S. X. L., Y. L. C., and S. T. L. reviewed the data and contributed to the drafting of the manuscript. T. T. approved the final content of this manuscript.

## ETHICS STATEMENT

All procedures performed were in accordance with the ethical standards of the institution and/or national research committee, in accordance with the 1964 Helsinki declaration and its later amendments or comparable ethical standards. Informed consent was obtained from all individual participants included in the study.

## Data Availability

The data that support the findings of this study are available from the corresponding author upon reasonable request. All data supporting the results reported can be provided by the corresponding author.
